# Seasonal variations in C/N/P/K stoichiometric characteristics in different plant organs in the various forest types of Sygera Mountain

**DOI:** 10.3389/fpls.2024.1293934

**Published:** 2024-02-01

**Authors:** Yueyao Li, Jiaxiang Wang, Luqi Wang

**Affiliations:** ^1^ Institute of Tibet Plateau Ecology, Tibet Agricultural & Animal Husbandry University, Nyingchi, China; ^2^ School of Chemical and Environmental Engineering, Wuhan Polytechnic University, Wuhan, China; ^3^ Anhui Agricultural University, Anhui, China

**Keywords:** Sygera Mountain, organs, forest type, season, stoichiometry

## Abstract

We explored the resource acquisition and growth strategies of plants adapting to different environments, focusing on the typical forest types of Sygera Mountain: *Pinus armandii*, *Picea likiangensis* var. *Linzhiensis*, *Abies georgei* var. *Smithii*, and *Juniperus saltuaria*. Then, we analyzed the nutrient content and stoichiometric ratios of C, N, P, and K in different plant organs (leaves, branches, trunks, and roots) to examine the stoichiometric characteristics and nutrient balance mechanisms in these forests. Results show that within the same forest type, different plant organs exhibit high C and low N, P, and K levels. N content in all organs followed the order leaves > branches > roots > trunks. During the growth phase, the concentrations of P and K in PLL and AGS follow the order branches > leaves > roots > trunks. In the dormant phase, the distribution in different organs had the order leaves > branches > roots > trunks. C content remained relatively stable over time. In the same organ across different forest types, increase in nitrogen content in plant leaves is an active adaptation of JS plants, indicating that JS has a conservative growth strategy and can adapt to environmental stress. Owing to the influence of seasons, the evolution process of N and P content fluctuates, allocating nutrients to supporting and transporting organs for resource optimization and allocation. The N and P content were lower in the growth phase than in the dormant phase. Seasonal variations in the C/N, C/P, and C/K ratios in different forests were inversely correlated with changes in N, P, and K content in plant organs, supporting the “growth rate hypothesis.” Stoichiometric analysis suggests that different limiting elements exist in organs across various forest types. Principal component analysis indicates that the seasonal patterns of stoichiometric ratios in the organs of different forest types show species-specific characteristics, reflecting the evolutionary nutrient utilization strategies of plant genera. In summary, plant growth in different Sygera Mountain forest types is limited by N and P, with a high tendency toward nitrogen limitation. The nutrient utilization and distribution differences among various organs during different growth stages are primarily influenced by the limited availability of environmental nutrients and inherent physiological characteristics of the plants.

## 1 Introduction

Ecological stoichiometry an invaluable approach for investigating the balance of nutrient elements in plants, facilitating for the systematic control of nutrient proportions within ecosystems (Yan et al., 2021). Its application allows the identification of interactions and constraints among elements. C serves as the fundamental structural material constituting plant skeletons, and N, P, and K are the key components of various proteins and genetic materials (Rong et al., 2015). They are essential to the identification of vegetation limitation type, and C/N, C/P, and C/K ratios are indicators of plant growth rates and efficiency with which plants utilize N, P, and K (Wassen et al., 1995; Agüero et al., 2014). Plant elemental composition considerably varies due to genetic characteristics, environmental conditions, and developmental stages. Therefore, in investigation into the nutrient content and ratios within plants contributes to the understanding of the structure and function of ecosystems and resource limitation conditions.

Plants employ different resource utilization strategies at various growth stages, and the stoichiometric characteristics of C, N, P, and K in different organs fluctuates throughout growth phases (Xiong et al., 2020; Liu et al., 2015). However, current research mainly focuses on the analysis of nutrient elements in litter on a stand scale (Deng et al., 2023; Liu et al., 2023), the distribution patterns and environmental drivers of nutrient content in plant leaf organs, and the interannual variability or multiyear averages of leaf nutrient stoichiometry (Ma et al., 2023; Yan et al., 2021; Li et al., 2023). Studies on the stoichiometric characteristics of branches, roots, and trunks are limited, overlooking the seasonal dynamics of elemental stoichiometry in leaf organs and other organs. In fact, different plant organs play distinct roles in survival mechanisms. For example, leaf organs primarily carry out photosynthesis and transpiration, branch organs provide support and conductance, root organs are important for nutrient absorption and support, and trunk organs are involved in transport and storage. Organs with high activity have substantial N, P, and K requirements, suggesting that nutrients are preferentially distributed to these organs. Seasonal fluctuations in the stoichiometric characteristics of different organs may increase the uncertainty of analysis results. Ren et al. (2007) and Sardans et al. (2012) compared the ecological stoichiometric characteristics of plant types to determine ecological strategies and environmental adaptation mechanisms; they found that ecological stoichiometry varies among tree species. Zhao et al. (2014) observed that nutrient distributions in different organs reflect a plant’s long-term response to environmental variations because different organs play distinct roles in growth and development and thus have varying nutrient demands and distribution. Yan et al. (2013) showed that growth in hickory leaves does not considerably vary between July and September, and root growth remains consistent throughout the growth phase, indicating seasonal dynamics in elemental distribution within hickory plants. In conclusion, plant growth in different seasons is influenced by environmental factors and internal regulatory mechanisms, and seasonal variations in temperature, moisture, nutrient supply, and internal hormones affect growth and development to varying degrees (Bai et al., 2016). Therefore, studying plant growth characteristics and the interactions between plants and their environment is crucial for understanding nutrient utilization strategies in different seasons and conditions.

Sygera mountain National Forest Park is located in the southeastern region of Tibet (Xin et al., 2004), and is an important component of the state-owned forest area in southeastern China. It plays an important role in regional climate and water source, soil, and water conservation. In view of this, this study selected four different forest types in Sygera mountain: *Pinus armandii* (PA), *Picea likiangensis* var. *Linzhiensis* (PLL), *Abies georgei* var. *Smithii* (AGS), and *Juniperus saltuaria* (JS). Seasonal variations in the stoichiometric characteristics of C, N, P, and K in various plant organs and their correlation with environmental factors. The following question was raised: Is there any trade-off mechanism between different elements in different organs of plants during seasonal changes (i.e., elements preferentially absorbed, the reabsorption efficiency of different nutrients, and the need to meet plant growth and stoichiometric component balance).

## 2 Materials and methods

### 2.1 Overview of the study area

**Figure 1:**
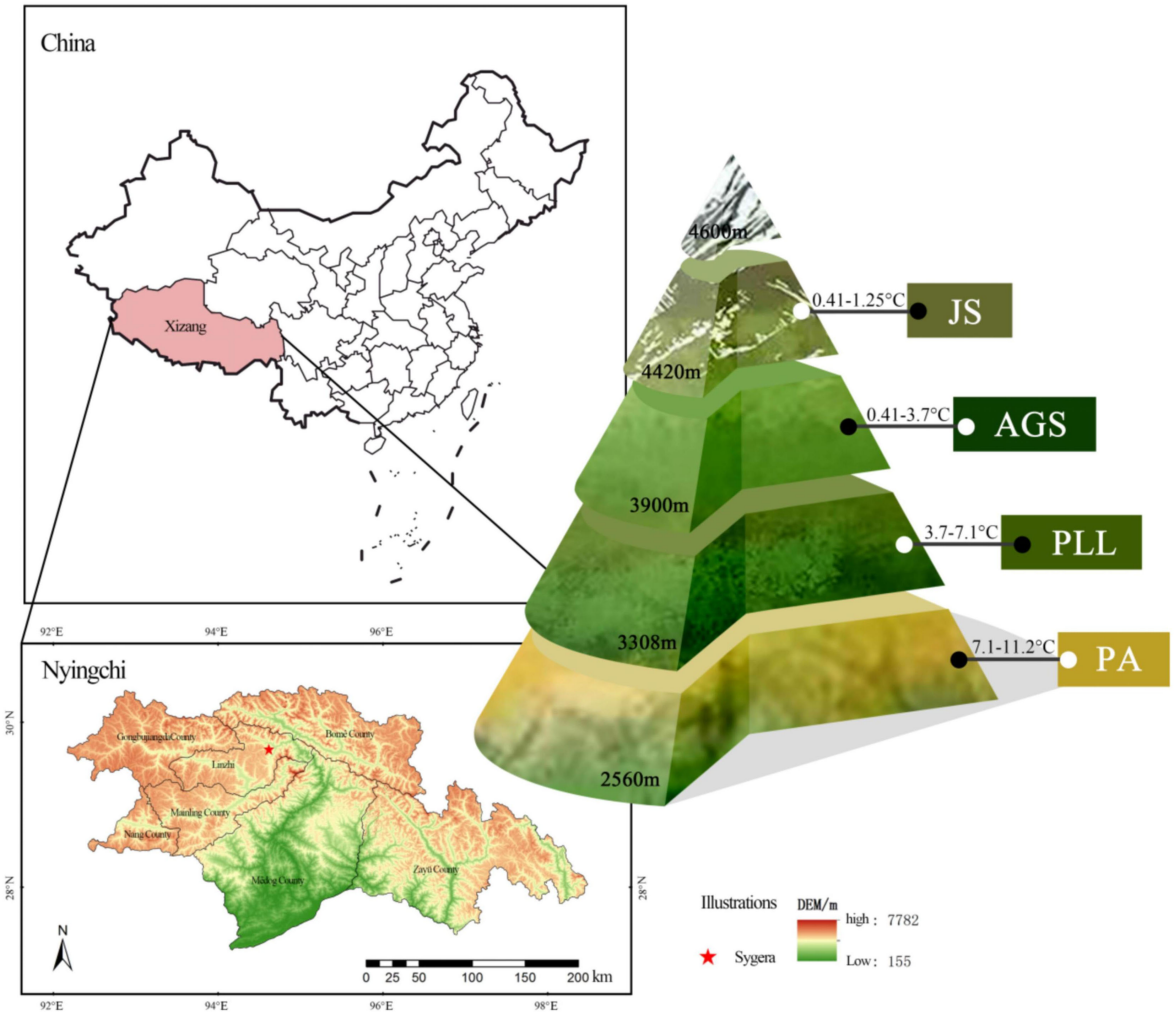
Distribution of different forest types in Sygera Mountain.

The Sygera Mountain, located within the territory of Nyingchi City in southeastern Tibet (94°12′–35′ E, 29° 15′–50′ N), forms part of the Nyainqêntanglha Mountain Range. It serves as the watershed between the Nyang River and Parlung Tsangpo. The main peak reaches an approximate elevation of 5300 m. The terrain is rugged and complex with a distinct vertical zonation. The area is characterized by a rich variety of vegetation types, minimal human disturbance, and a forest coverage rate of 56%. It falls within the subalpine cold temperate humid climate zone, and the annual average temperature is approximately −0.73°C. The warmest month (July) has an average temperature of 9.23°C, whereas the coldest month (January) has an average temperature of −13.98°C. The rainy season spans from June to September, accounting for 75%–82% of the annual precipitation. The area receives an average annual rainfall of 1134 mm and has an average annual relative humidity of 78.83%. The frost-free period lasts 178 days, and the total annual sunshine duration is approximately 1150 hours. The forest distribution line is over 4400 m and classified as an alpine cold zone. At 3900 m, it is a subalpine cold temperate zone; 3300 m marks the mountain temperate zone in river valleys, and the warm and temperate humid subvalleys of the mountain are below 2500 m (**Figure 1**). The soil is predominantly mountain brown earth. The study area has a rich variety of vegetation and serves as a core area for biodiversity conservation in southeastern Himalayas. It is a crucial component of Tibet’s ecological security barrier and a “natural laboratory” for studying vegetation changes.

### 2.2 Sample plot settings

The forest communities in southeastern Tibet are predominantly mountain forests, exhibiting significant spatial heterogeneity. To minimize potential impacts arising from topographic conditions, we selected four typical forest types (PA, PLL, AGS, JS) from stands with minimal human disturbances, such as grazing, logging, or fires, according to comprehensive surveys. The selection was in line with the community structure distribution characteristics of the Sygera Mountain. One fixed 30 m × 30 m plot was established in each forest type. Within these plots, a detailed survey of trees was conducted, including measuring every tree and mapping their locations. Key metrics, such as the coordinates, diameter at breast height, and height of all target plants within the plots, were recorded. The basic details are presented in **Table 1**.

**Table 1:** Basic characteristics of four typical forest types of plants in Sygera.

Elevation(m)	Latitude and longitude	Forest type	Slope (°)	CD (%)	TH (m)	DBH (cm)	MAT (°C)	Type	Major understory species
2560	94°47′51″E 29°56′49″N	PA	33	50	26.0 ± 0.8	128.4 ± 4.3	7.10~11.20	E-N-A	Cupressus gigantea、Pinus densata、Rosa sericea Lindl.f.pteracantha Franch.、
3308	94°43′~34′E 29°41′~56′N	PLL	7	40	20.8 ± 0.7	156.2 ± 6.8	3.70~7.10	E-N-A	Betula utilis、Quercus aquifolioides、Larix gmelinii、Abies georgei var. Smithii、Sinarundinaria setosa Yi
3900	94°42′~54′E 29°39′~1′N	AGS	32	80	21.4 ± 1.4	177.4 ± 7.2	0.41~3.70	E-N-A	Spiraea schneideriana、Sorbus rehderiana
4420	94°41′~45′E 29°36′~51′N	JS	25	50	10.2 ± 0.7	90.8 ± 4.5		E-N-A	Rhododendron pingianum、Salix lindleyana、Rhodiola fastigiata、Rh. aganniphum Balf.f.et Ward

### 2.3 Sample collection and analysis

In July–August 2022 (growth phase) and November–December 2022 (dormant phase), sampling was conducted across various forest types. Within each site, five standard trees, characterized by good health, uniform size, and complete morphological structure, were selected from different tree species for sampling.

For the leaf and branch organs: pruning shears were used to gather leaves (1–3 years old) and branches (1–2 years; old new growth) from the middle canopy in all four cardinal directions (east, west, north, and south) of each plant. The samples were then mixed proportionally to represent individual leaf and branch specimens.

For the trunk organs, a 5 mm increment borer was utilized to extract five cores reaching the pith from the standard trees at 1.3 m breast height. The samples were mixed. The boreholes in the trees were subsequently filled with butter to prevent pathogen intrusion.

For root organs: The “excavation method” was employed to collect root systems from a soil depth of 0–30 cm. After washing, fine roots smaller than 2 mm were selected as tree root samples and placed in ziplock bags, labeled, and refrigerated for preservation. The samples were subdivided, cleaned, and subjected to other preparatory treatments. They were initially heated in an oven at 105°C for 15 min, dried at 80°C to constant mass, ground, and sieved through a 1 mm mesh. Finally, the samples were bagged, sealed, and stored for subsequent analysis.

### 2.4 Chemical analysis of plant nutrient elements

Elemental Analysis: The C and N content in plant samples were measured using an elemental analyzer (vario EL cube CHNOS Elemental Analyzer, Elementar Analysensysteme GmbH, Germany). The P and K content were determined through HNO_3_–H_2_O_2_ digestion and ICP-OES (iCAP 6300 ICP-OES Spectrometer, Thermo Fisher, USA). Meteorological data were derived from long-term observations at three different altitudes: 3500, 3900, and 4300 m. Given the absence of meteorological data at 2500 m, we selected meteorological data at altitudes of 3500, 3900, and 4300 m for principal component analysis (PCA) in correlation with the nutrient content of corresponding forest plants to ensure the uniformity of meteorological instrument performance. The meteorological instruments were installed within the forest, and the system was programmed to automatically calculate and store the observed meteorological data every 10 min. Data loggers were produced by Onset Company, USA (HOBO H21-USB).

### 2.5 Data processing

The preliminary processing of data from four typical forest ecosystem types in Sygera Mountain was conducted using WPS-Excel 2022. Statistical analyses were performed using SPSS 25.0, and graphics were created with Origin 2021. Multifactorial analysis of variance and Duncan’s multiple range test were employed to compare the nutrient content and stoichiometric ratios of different forest types and organs, and the significance level was set at 0.05. To further analyze the characteristics of each element, we used online tools (https://www.bioinformatics.com.cn) for graphical representation. PCA was applied to explore the correlations among environmental factors, nutrient content, and stoichiometric ratios in the plant organs of different forest stands and identify elements and stoichiometric ratios with high loadings on the PCA axes.

## 3 Results

### 3.1 Decomposition of plant C/N/P/K stoichiometric variability

The influence of organ, forest type, season, and their interactions on the content of C, N, P, and K in plants and their stoichiometric ratios vary (**Table 2**). The C content in plants is primarily affected by the interaction between organ and forest type, and the highest Type IIISS reaches 16,950.89. However, the impact of forest type on C content is not significant (*p* > 0.05), and season has the least influence (Type IIISS: 0.12). N, P, and K content are mainly influenced by organ. Subsequently, the interaction between the organ and season has the least impact on the N and P content (Type IIISS: 2.19, 0.03), whereas the influence of season on K content is minimal.

**Table 2:** Analysis of variation sources of C, N, P, and K content in different forest types.

Parameters	Item	Source of variation					
		Organ (O)	Forest type (F)	Season (S)	O×F	O×S	O×F×S
C	Type IIISS	13087.11	1218.14	0.12	16950.89	201.12	3923.36
	DF	3.00	3.00	1.00	9.00	3.00	9.00
	MS	4362.37	406.05	0.12	1883.43	67.04	435.93
	F	25.21	2.35	0.00	10.88	0.39	2.52
	P	0.00	0.08	0.98	0.00	0.76	0.01
N	Type III SS	2646.40	47.83	2.76	29.88	2.19	30.34
	DF	3.00	3.00	1.00	9.00	3.00	9.00
	MS	882.13	15.94	2.76	3.32	0.73	3.37
	F	1159.32	20.95	3.63	4.36	0.96	4.43
	P	0.00	0.00	0.06	0.00	0.42	0.00
P	Type III SS	30.11	2.02	0.04	4.20	0.03	0.72
	DF	3.00	3.00	1.00	9.00	3.00	9.00
	MS	10.04	0.67	0.04	0.47	0.01	0.08
	F	278.43	18.71	1.12	12.94	0.28	2.22
	P	0.00	0.00	0.29	0.00	0.84	0.02
K	Type III SS	426.42	71.50	0.99	93.21	4.29	23.72
	DF	3.00	3.00	1.00	9.00	3.00	9.00
	MS	142.14	23.83	0.99	10.36	1.43	2.64
	F	149.96	25.14	1.05	10.93	1.51	2.78
	P	0.00	0.00	0.31	0.00	0.22	0.01

*Type IIISS is the sum of squares of deviations, and the greater the value, the stronger the factor’s influence is.

Variability in C/N, C/P, and C/K ratios is primarily influenced by the type of organ (Type IIISS: 2362299.26, 197740451.69, 4698339.61; **Table 3**), followed by the interaction between organ and forest type (Type IIISS: 260245.40, 51934754.08, 1675459.18). Forest type has the highest influence on variations in N/P and N/K ratios (Type IIISS: 223.62, 30.76), whereas season has the least. The influence of season and the interaction between organ and season on the N/P and N/K ratios is not significant (*p* > 0.05). Variability in K/P ratio is mainly influenced by organ (Type IIISS: 92.13), followed by forest type and their interaction.

**Table 3:** Sources of variation in ecological stoichiometry among different forest types.

Parameters	Item	Source of variation					
		Organ (O)	Forest type (F)	Season (S)	O×F	O×S	O×F×S
C/N	Type IIISS	2362299.26	142505.10	29998.35	260245.40	78500.00	63388.96
	DF	3.00	3.00	1.00	9.00	3.00	9.00
	MS	787433.09	47501.70	29998.35	28916.16	26166.67	7043.22
	F	234.16	14.13	8.92	8.60	7.78	2.09
	P	0.00	0.00	0.00	0.00	0.00	0.04
C/P	Type III SS	197740451.69	17556522.29	8658916.64	51934754.08	24856194.31	33642818.27
	DF	3.00	3.00	1.00	9.00	3.00	9.00
	MS	65913483.90	5852174.10	8658916.64	5770528.23	8285398.10	3738090.92
	F	30.51	2.71	4.01	2.67	3.84	1.73
	P	0.00	0.05	0.05	0.01	0.01	0.09
C/K	Type III SS	4698339.61	414304.84	117811.49	1675459.18	399234.40	842063.52
	DF	3.00	3.00	1.00	9.00	3.00	9.00
	MS	1566113.20	138101.61	117811.49	186162.13	133078.13	93562.61
	F	33.34	2.94	2.51	3.96	2.83	1.99
	P	0.00	0.04	0.12	0.00	0.04	0.05
N/P	Type III SS	113.66	223.62	7.68	185.28	18.01	62.77
	DF	3.00	3.00	1.00	9.00	3.00	9.00
	MS	37.89	74.54	7.68	20.59	6.00	6.97
	F	15.83	31.14	3.21	8.60	2.51	2.91
	P	0.00	0.00	0.08	0.00	0.06	0.00
N/K	Type III SS	26.86	30.76	0.11	11.58	0.51	3.63
	DF	3.00	3.00	1.00	9.00	3.00	9.00
	MS	8.95	10.25	0.11	1.29	0.17	0.40
	F	61.75	70.73	0.72	8.87	1.16	2.78
	P	0.00	0.00	0.40	0.00	0.33	0.01
K/P	Type III SS	92.13	47.98	10.06	37.48	5.79	3.05
	DF	3.00	3.00	1.00	9.00	3.00	9.00
	MS	30.71	15.99	10.06	4.16	1.93	0.34
	F	33.51	17.45	10.97	4.54	2.11	0.37
	P	0.00	0.00	0.00	0.00	0.10	0.95

* Type IIISS is the sum of squares of deviations, and the greater the value, the stronger the factor’s influence is.

Different forest types exhibit varying characteristics of seasonal variability in C, N, P, and K content and their stoichiometric ratios in various plant organs (**Table 4**). Different forest types have small coefficient of variation (CV) for C content (0.92%–3.33%), indicating weak variability. Throughout the growth phase, the highest CVs for N content are found in the trunk organs in PA and PLL (26.53% and 24.42%, respectively), indicating moderate variability. The highest CV for P content is found in the trunk organs in JS (48.45%), indicating high variability. As for K content, the highest CV is found in root organs in AGS (39.55%), also indicating high variability. The lowest CVs for N, P, and K content are found in branch organs in JS (2.74%, 3.86%, and 5.79%, respectively), indicating weak variability. The highest CVs for C/N, C/P, C/K, N/P, and N/K ratios are found in trunk organs in PA, ranging from 36.27% to 92.12% and indicating high variability. By contrast, the highest CV for K/P ratio is found in trunk organs in PLL (26.66%), indicating moderate variability. During the dormant phase, the highest CVs for N and P content in trunk organs are found in JS indicate moderate variability (31.63%) and high variability (38.66%), respectively. The highest CV for K content is obtained in root organs in PA (46.60%), indicating high variability. The highest CVs for C/N and C/P ratios are found in trunk organs in JS (33.30% and 39.03%, respectively). As for C/K ratio, the highest CV is found in root organs in PA (29.83%), and lowest is found in branch organs in JS (6.19%). The highest CVs for N/P and N/K ratios are found in root organs in PA (21.62% and 31.24%, respectively), indicating moderate variability. The highest CV for K/P ratio is found in trunk organs in PLL (32.62%), indicating moderate variability. The lowest CV is found in root organs in JS (3.58%), indicating weak variability. (CV ≤ 15% indicates weak variability; 15% < CV ≤ 35%, moderate variability; 35% < CV ≤ 100%, high variability; CV ≥ 100%, strong variability; Kobierski et al., 2014).

**Table 4:** Coefficient of variation between different forest types and organs (%).

Time	Forest type	Organ	C	N	P	K	C/N	C/P	C/K	N/P	N/K	K/P
Growth phase	PA	L	0.35	7.67	10.06	15.52	7.68	10.89	17.52	6.75	11.78	7.94
		B	1.10	12.44	13.54	12.11	11.86	12.07	12.66	7.99	12.61	10.81
		T	10.40	26.53	37.70	35.47	40.22	92.12	83.25	43.53	36.27	8.41
		R	1.46	7.44	11.34	22.50	6.54	11.33	21.72	14.70	21.65	14.37
		Mean	3.33	13.52	18.16	21.40	16.58	31.60	33.79	18.24	20.57	10.38
	PLL	L	1.12	7.77	15.36	10.68	6.87	14.02	10.41	16.40	11.55	17.57
		B	0.70	12.76	11.40	14.03	14.32	11.12	13.16	6.50	16.99	12.65
		T	4.33	24.42	44.35	32.19	21.27	33.60	35.70	19.46	31.64	26.66
		R	2.79	22.96	36.15	25.32	19.93	34.07	24.95	28.61	19.72	24.74
		Mean	2.23	16.98	26.82	20.55	15.60	23.20	21.06	17.74	19.97	20.40
	AGS	L	0.83	7.37	8.15	23.85	7.57	8.96	35.11	3.18	26.93	19.33
		B	1.33	12.75	17.61	20.99	15.02	22.66	30.04	9.38	13.64	8.21
		T	0.25	10.25	33.55	36.50	9.16	24.71	33.44	17.64	26.81	14.31
		R	1.48	6.71	47.32	39.55	7.10	31.11	37.09	30.63	34.82	25.50
		Mean	0.97	9.27	26.66	30.22	9.71	21.86	33.92	15.21	25.55	16.84
	JS	L	0.85	4.19	4.00	12.18	3.64	3.67	11.91	3.45	9.40	12.90
		B	0.87	2.74	3.86	5.79	3.58	4.36	6.05	3.09	4.71	6.30
		T	0.47	20.10	48.45	27.06	18.99	37.17	22.76	22.60	8.39	17.36
		R	1.72	9.46	16.52	27.92	8.66	16.78	24.86	9.32	17.63	11.70
		Mean	0.98	9.12	18.21	18.24	8.72	15.49	16.40	9.62	10.03	12.07
Dormant phase	PA	L	0.57	8.16	6.95	10.65	8.38	7.15	10.47	12.24	8.51	9.39
		B	1.05	18.23	16.14	15.35	16.14	14.74	15.83	7.22	11.61	4.78
		T	1.53	17.06	11.09	12.63	15.82	12.59	12.24	11.29	27.25	20.17
		R	1.38	7.01	32.48	46.60	8.38	24.09	29.83	21.62	31.24	16.60
		Mean	1.13	12.61	16.67	21.31	12.18	14.64	17.09	13.09	19.65	12.73
	PLL	L	0.70	6.46	13.47	13.19	6.36	14.85	12.72	18.56	14.35	17.67
		B	1.32	15.38	17.54	18.75	16.58	19.07	15.78	7.39	17.13	12.79
		T	0.59	8.62	18.74	20.65	9.40	16.12	19.61	12.81	22.30	32.62
		R	1.34	16.72	33.98	28.92	17.61	32.39	28.34	17.49	21.00	17.20
		Mean	0.99	11.80	20.93	20.38	12.49	20.61	19.11	14.06	18.70	20.07
	AGS	L	0.73	6.09	7.58	15.01	6.47	7.94	17.64	5.20	13.29	11.62
		B	1.39	13.01	12.44	17.28	13.41	14.11	19.37	4.54	11.03	7.84
		T	0.87	26.92	31.57	34.00	28.54	27.66	29.77	8.42	14.22	10.60
		R	1.10	8.89	10.31	17.85	9.85	10.16	18.43	13.08	26.65	22.09
		Mean	0.92	5.97	7.27	9.31	14.57	14.97	21.30	7.81	16.30	13.04
	JS	L	0.92	5.97	7.27	9.31	6.55	7.56	9.38	4.87	7.99	8.82
		B	1.03	5.32	3.76	5.44	4.56	3.42	6.19	6.35	10.77	5.92
		T	1.60	31.63	38.66	23.71	33.30	39.03	23.28	7.05	20.17	28.47
		R	2.41	12.84	17.75	14.93	14.26	21.35	18.32	10.97	8.54	3.58
		Mean	1.49	13.94	16.86	13.35	14.67	17.84	14.29	7.31	11.87	11.70

* *PA, Pinus armandii; PLL, Picea likiangensis var. Linzhiensis; AGS, Abies georgei var. Smithii; JS, Juniperus saltuaria*.

### 3.2 Stoichiometric characteristics of different organs in the same forest type

**Figure 2:**
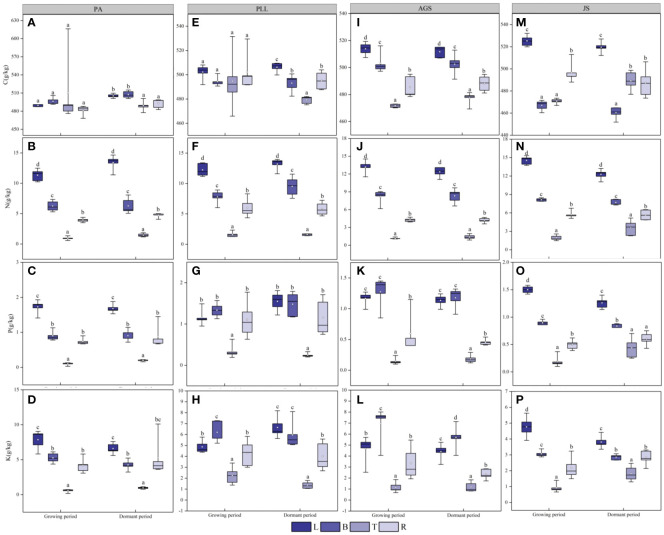
Seasonal dynamics of C, N, P, and K content in different organs of the same forest. type. L, leaf; B, branches; T, trunk; R, roots, PA, *Pinus armandii*; PLL, *Picea likiangensis* var. *linzhiensis*; AGS, *Abies georgei* var. *smithii*; JS, *Juniperus saltuaria*. **(A-P)** represents the seasonal variation characteristics of C, N, P, and K nutrient content in various organs of different forest types. Different lowercase letters represent significant differences among different organs of the same forest type (*p* < 0.05). Same below.

During the growth phase (**Figure 2**), the C content in trunk organs in PA is significantly higher than that in leaves, branches, and root organs. In PLL, AGS, and JS, the highest C content is observed in leaf organs. The highest N content is found in leaf organs, whereas the lowest in trunk organs in all forest types, and both show a consistent distribution sequence: leaf > branch > root > trunk organ. The distribution patterns of P and K are similar, showing the lowest nutrient content in trunk organs. However, in PLL and AGS, branch organs have higher P content (1.34 and 1.27 g/kg, respectively) and K content (6.21 and 6.95 g/kg, respectively) than leaf organs. In PLL, AGS, and JS, leaf organs show the highest C content during the dormant phase. In the growth phase, the highest C content is found in branch organs in PA. Except the P and K content in AGS branches, all other forest types show the highest N, P, and K content in leaf organs, with the smallest amounts in trunk organs, following the sequence leaf > branch > root > trunk organ. Except for the insignificant difference in C content among different organs in PA and PLL forests during the growth phase (*p* > 0.05), significant differences in elemental content were observed in the organs of other forest types (*p* < 0.05).

**Figure 3:**
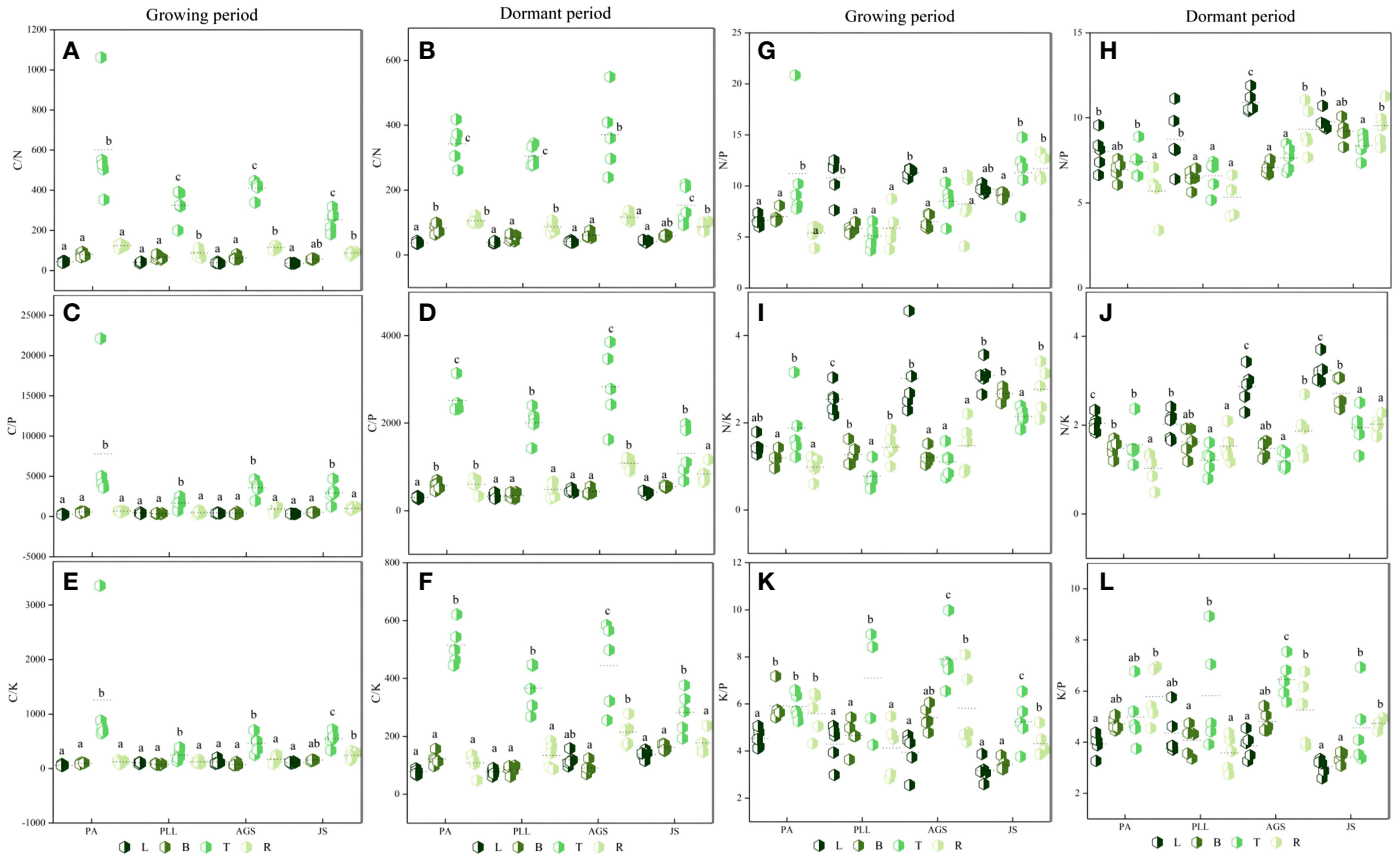
Seasonal dynamics of ecological stoichiometry in different organs of the same forest type. L, leaf; B, branches; T, trunk; R, roots; PA, *Pinus armandii*; PLL, *Picea likiangensis* var. *linzhiensis*; AGS, *Abies georgei* var. *smithii*; JS, *Juniperus saltuaria*. **(A–L)** represents the seasonal variation characteristics of the ecological stoichiometry of different organs in the same forest type. Different lowercase letters represent significant differences among different organs of the same forest type (*p* < 0.05).

During the dormant phase (**Figure 3**), the C/N, C/P, and C/K ratios in various forest types are 36.17– 600.09, 286.68– 7800.10, and 63.93– 1262.63, respectively, and those in trunk organs are significantly higher than those in leaves, branches, and roots. In PA and JS, the smallest ratios are observed in leaf organs, and the order of organ sequence based on C/N, C/P, or C/K ratio is trunk > root > branch > leaf (**Figures 3A, C, E**). In PLL and AGS, this pattern is inconsistent, and no significant differences (*p* > 0.05) in C/N and C/P ratios are found among the leaves, branches, and roots in PA, PLL, and AGS. Except in PA and JS, the highest N/P ratios are found in leaf organs and exhibit significant differences (*p* < 0.05). Leaf organs have the smallest K/P ratios in all forest types, except PLL. In the dormant phase, the trunk organs have the highest C/N, C/P, and C/K ratios in all forest types (**Figures 3B, D, F**). Except the C/P and C/K ratios in AGS, the smallest values are observed in leaf organs. Conversely, leaf organs have the highest N/P and N/K ratios in all forest types. The K/P ratio does not follow a uniform pattern of change. Seasonal variations significantly affect the elemental content in various organs in all forest types (*p* < 0.05).

### 3.3 Stoichiometric characteristics in the same organ in different forest types

**Figure 4:**
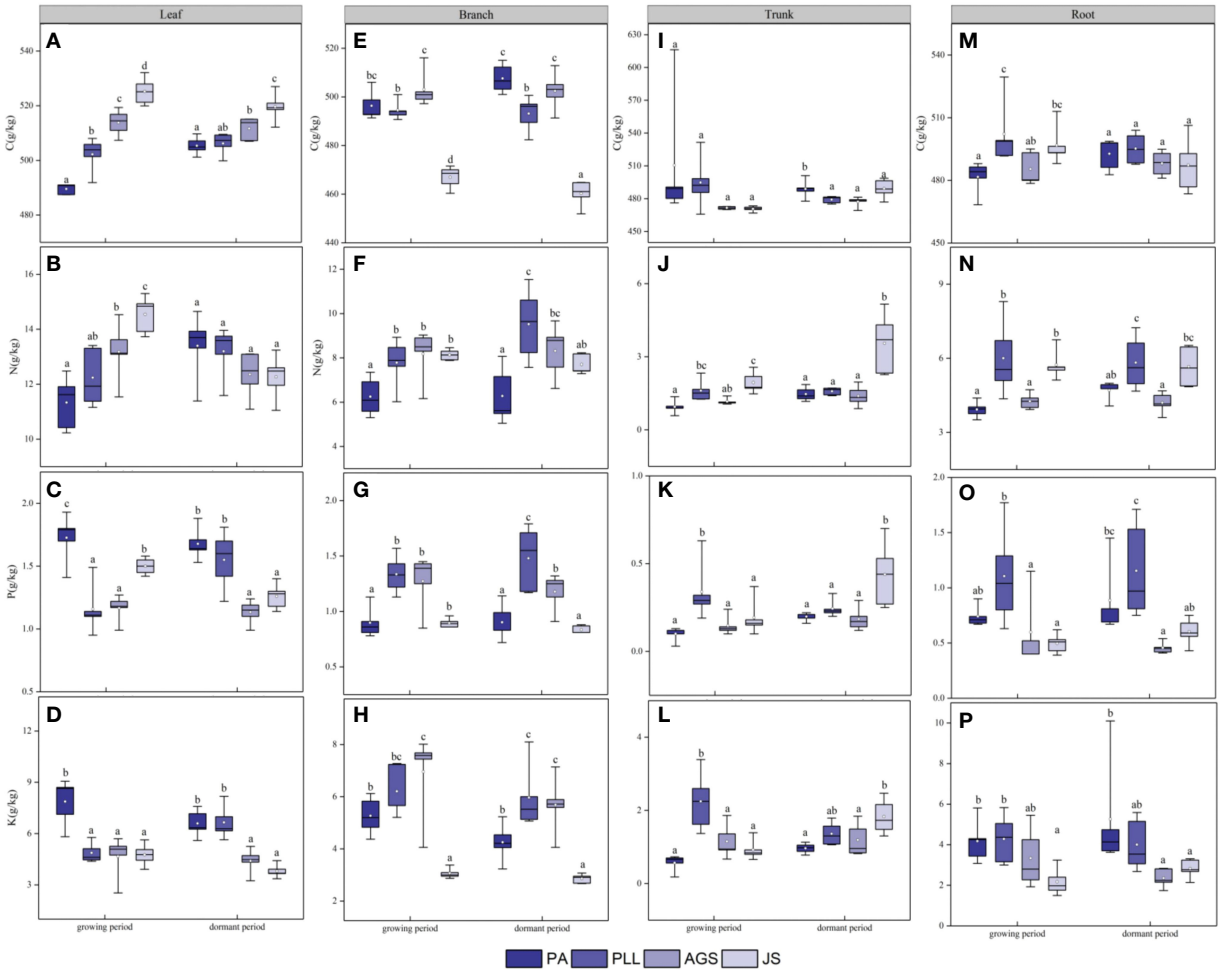
Seasonal Dynamics of C, N, P, K Content in Different Forest Types of the Same Organ. PA: *Pinus armandii*; PLL: *Picea likiangensis* var. *linzhiensis*; AGS: *Abies georgei* var. *smithii*; JS: *Juniperus saltuaria*. **(A–P)** represents the seasonal dynamics of nutrient content in different forest types within the same organ. Different lowercase letters represent significant differences in different forest types of the same organ (*p* < 0.05).

In the growth phase (**Figure 4**), leaf organs in JS exhibit significantly higher C and N content than those in the other three forest types, and PA has the lowest C and N content (11.33 and 489.56 g/kg, respectively). The order of C and N content from highest to lowest is as follows: JS > AGS > PLL > PA. Conversely, the PA forest had the highest P and K content (1.73 and 7.87 g/kg, respectively), showing significant differences (*p* < 0.05). In the branch organs, PLL has the highest P content. The highest C, N, and K content are found in the AGS forest, measuring 6.95, 8.18, and 503.07 g/kg, respectively. Notably, the differences in N content among PLL, AGS, and JS are not significant (*p* > 0.05; **Figure 4F**). In the trunk organs, no significant difference in C content is found among the forest types (*p* > 0.05); the lowest N, P, and K content are found in PA (**Figures 4J, K, L**). In the root organs, the highest C and N content are observed in PLL (6.01 and 502.20 g/kg, respectively), and the lowest values are found in PA. The order of the forest types by C and N content is PLL > JS > AGS > PA. The highest P and K content are found in the root organs in PLL, but the order of the forest type sequence is PLL > PA > AGS > JS. In the dormant phase, the C content in the leaf organs is significantly higher in JS than in the other three types, showing a significant difference (*p* < 0.05). The N content exhibits an inverse state, with no significant differences observed (*p* > 0.05); the P and K content show no clear pattern of variation among different forest types. The highest carbon content in branch organs is observed in AGS (502.46 g/kg). PLL has the highest N content (9.52 g/kg). The order of the forest type sequence according to P and K content in the branch organs is PLL > AGS > PA > JS (**Figures 4G, H**), and differences are significant (*p* < 0.05). In the trunk organs, JS consistently shows the highest values, and the N and P content have the following order: JS > PLL > PA > AGS. No significant difference in carbon content in the root organs is found among the forest types (*p* > 0.05), but the highest N, P, and K content are observed in AGS (1.16, 5.26, and 5.82 g/kg, respectively), and values exhibit significant differences (*p* < 0.05).

**Figure 5:**
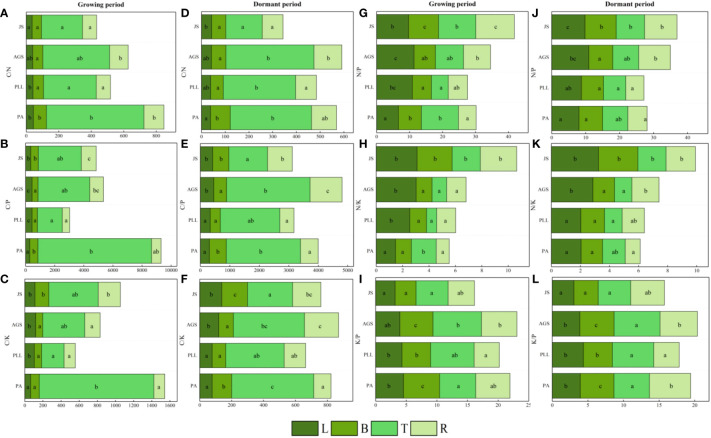
Seasonal dynamics of ecological stoichiometry in different stand types of the same organ. L, leaf; B, branches; T, trunk; R, roots; PA, *Pinus armandii;* PLL, *Picea likiangensis* var. *linzhiensis*; AGS, *Abies georgei* var. *smithii*; JS, *Juniperus saltuaria*. **(A–L)** represents the seasonal dynamics of the ecological stoichiometry of different forest types in the same organ. Different lowercase letters represent significant differences in different forest types of the same organ (*p* < 0.05).

In the growth phase (**Figure 5**), the leaf organs of PA exhibit significantly higher C/N ratios than those in the other three forest types, and the lowest ratio is found in JS (36.17). The order is PA > PLL > AGS > JS, and significant differences are observed. The highest C/P and C/K ratios are found in AGS (444.23 and 120.18, respectively); the N/P ratio has the following order: AGS > PLL > JS > PA. The highest N/K ratio in leaf organs is observed in JS, following the order JS > AGS > PLL > PA. No significant differences are found among JS, GA, and PLL; the K/P ratio is inversely related and has the order PA > PLL > AGS > JS. The highest C/N ratio in branch organs is found in PA, and no significant differences are found among the other forest types (*p* > 0.05); the highest C/P and K/P ratios in the branch organs are found in PA (562.78 and 5.91, respectively), and significant differences are observed (*p* < 0.05). The highest C/K, N/P, and N/K ratios in the branch organs are observed in JS (2.66, 9.10, and 153.10, respectively). With regard to trunk organs, the PA forest exhibit significantly higher C/N, C/P, and C/K ratios than the other three forest types; the highest N/P and N/K ratios in trunk organs are found in JS (2.14 and 11.32, respectively), and the N/P ratios follows the order JS > PA > AGS > PLL (**Figures 5G, I**), and the highest K/P ratio is observed in AGS, and the lowest is found in JS (5.23), and significant differences are found among the forest types (*p* < 0.05).

In the dormant phase, PA exhibits the lowest C/N and C/P ratios in leaf organs (37.98 and 302.47, respectively) and have the following sequences: JS > AGS > PLL > PA (C/N) and AGS > JS > PLL > PA (C/P). The highest C/K and N/K ratios in leaf organs are observed in JS, and both follow the order JS > AGS > PA > PLL. The highest N/P ratio in leaf organs is found in AGS and significantly differ from those of the other three forest types. The highest K/P ratio in JS is significantly higher than in the other forest types, and the order in descending K/P ratio is PLL > PA > AGS > JS. In the branch organs, the highest C/N and C/P ratios are observed in PA, and lowest is found in PLL; the highest C/K, N/P, and N/K ratios are found in JS, and the K/P ratio follows the order AGS > PA > PLL > JS. In the trunk organs, the order by C/N and C/P ratios have the following order: AGS > PA > PLL > JS; the order by C/K ratio is PA > AGS > PLL > JS; the highest N/P and N/K ratios are found in JS (1.94 and 8.36, respectively), and both show significant differences. The K/P ratio in the trunk organs show no significant difference among forest types, following the order AGS > PLL > PA > JS. In the root organs, the highest C/N, C/P, and C/K ratios are observed in AGS (117.18, 214.15, and 1084.65, respectively), and all show significant differences. The highest N/P and N/K ratios are found in JS, and the K/P ratio follows the order PA > AGS > JS > PLL.

### 3.4 PCA analysis of plant elemental content and stoichiometric ratios

**Figure 6:**
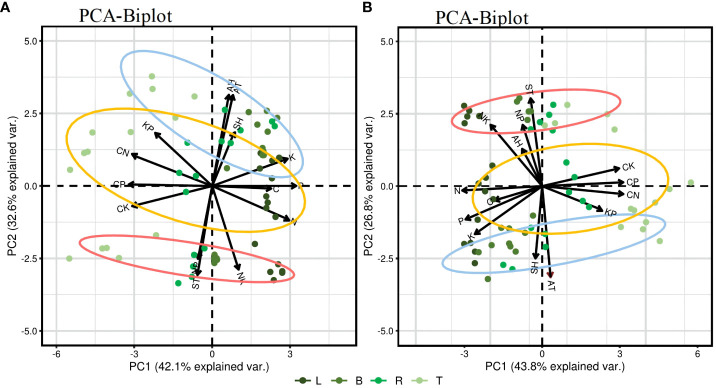
Principal component analysis (PCA) of environmental factors and the content and stoichiometric ratio of four elements in plant organs. In the biplot, horizontal and vertical axes (Ax1 and Ax2, respectively) denote the first and the second ordination axis of PCA, respectively. AT, air temperature; AH, air humidity; ST, soil temperature; SH, soil (10 cm) humidity. Blue circle, PLL forest; Yellow circle, AGS forest; Red circle, JS forest. **(A)** represents the PCA of the growth phase; **(B)** represents the PCA of the dormant phase.

**Figure 6** presents a PCA result of the C, N, P, and K content in different plant organs in relation to environmental factors. The influence of each parameter on the principal components is indicated by the length of arrows or lines. The angle between two parameters or between a parameter and the principal axis signifies the correlation among variables; a small angle indicates a strong correlation. On a seasonal scale, demonstrating a strong negative correlation among C/N, C/P, and C/K ratios and among the N, P, and K content and a strong positive correlation among N, P, and K content. In the PC1 factor loadings, nutrient contents, and stoichiometric ratios are distinctly separated along the PC1 axis, and the trends of C, N, P, K content are opposite to those of the C/N, C/P, and C/K ratios. PCA results for the growing phase show that the two principal components explain 74.7% of the total variance, and PC1 and PC2 account for 42.1% and 32.6%, respectively. AT, AH, and SH are highly positively correlated with C, P, and K content, and with the C/N and K/P ratios and negatively correlated with N content and the C/P and C/K ratios. ST shows a high positive correlation with N content, and the C/P, C/K, N/P, and N/K ratios; AT; AH; and SH are significant parameters affecting the stoichiometric ratios in the different organs of PLL, especially AT, which has a direct and significant effect. In the dormant phase (**Figure 6B**), the contribution rates of PC1 and PC2 are 43.8% and 26.8%, respectively, having a cumulative contribution of 69.6%. AT and SH show a high positive correlation with C, N, P, and K content and C/N, C/P, and K/P ratios and a negative correlation with the C/K, N/P, and N/K ratios. AH and ST are highly positively correlated with the N/P and N/K ratios, and ST is a significant parameter affecting stoichiometric ratios in the various organs of JS.

## 4 Discussion

### 4.1 Seasonal dynamics of C, N, P, and K contents of plant organs in different forest types

C, N, P, and K are indispensable elements in plants and are indicators of the plants’ capacities for nutrient absorption and storage in various habitats (Yu et al., 2014; Xu et al., 2016). A close relationship between the stability index and CV has been found in plants. A small CV in plant nutrient content indicates a high stability index. In this study, the leaf organs of all four forest types exhibit the smallest CV in C content during the growth and dormant phases (0.92%–3.33%; **Table 4**), suggesting a relatively stable level of C. This ability to regulate nutrient absorption, utilization, and storage in various organs aligns with the findings of Shi et al. (2018), reflects a “conservative” strategy in elemental content during the evolutionary process of organisms, and is in line with the stable leaf nutrient content hypothesis (Elser et al., 2000). Across different seasonal scales (**Figure 4**), leaf C content in different forest types range from 489.56 g/kg to 525.29 g/kg during the growth phase and 506.22– 519.60 g/kg during the dormant phase, significantly higher than the global plant nutrient level (464.00 g/kg; Elser et al., 2000; 446.04 g/kg; Zhang et al., 2022; 391.49 g/kg; Wu et al., 2023). The C leaf content in descending order is JS > AGS > PLL > PA in all forest types. A high leaf C content implies a low photosynthetic rate, slow growth rate, and strong defense against adverse external environments (Li et al., 2022), indicating that JS is the most resistant to adverse factors among the forest types. In the growth phase, the C content in the trunk organs shows a clear decreasing trend in all the forest types (**Figure 4I**), likely because these organs transfer energy for leaf growth and development and thereby decrease their own C content. The trend is slightly moderated during the dormant phase possibly because C accumulates through photosynthesis in leaves and is transported to the trunk. Thus, trunk C content increases and higher during the growth phase than in the dormant phase.

With seasonal changes, the N and P content (0.94– 14.54 g/kg for N and 0.10– 1.68 g/kg for P) of different forest types are lower than those at the global levels (20.60 and 1.99 g/kg, respectively; Elser et al., 2000) and also lower than those found in different study areas, such as the Three Gorges Reservoir region (Kong et al., 2020) and the Liangshan region in Sichuan (Yang et al., 2021). In areas with high precipitation, the degree of leaching of nitrate nitrogen in plant leaf organs is high, leading to a decrease in the mass fraction of N (Qi et al., 2018). Our study period coincides with the rainy season, a peak growth phase. The leaching of effective nitrogen alters nutrient content within plants, resulting in relatively low N and P content. Plants exhibit different levels of nutrient accumulation in various organs due to the distinct mechanisms of nutrient absorption and transport. The N content in the leaf organs of PA is lower than that in JS, and significant differences are observed in all organs, except in the leaves in the dormant phase (*p* < 0.05). The increase in leaf N content represents an active adaptation of plants in high- altitude forests. This effect enhances photosynthesis and mitigates the loss of organic C due to low-temperature stress. The leaves have the highest N and P content, and the other organs have higher N and P content in the dormant phase than in the growth phase. The N and P content have the following order: leaves > branches > roots > trunks. The N and P content in branch organs decrease after reaching their peak in the entire seasonal cycle (PLL). This phenomenon is primarily attributed to the high content of N-rich enzymes and P-rich RNA in plants growing in cold environments (Weih and Karlsson, 2001), leading to an increase in leaf N content in JS. This effect compensates for the reduced rate of biochemical reactions due to low temperatures, maintaining normal plant growth and aligning with the temperature–plant physiological hypothesis. This finding is consistent with the conclusions drawn from studies conducted in Qilian Mountains, Baotianman Mountain, and Helan Mountains by Zhang et al. (2022); Du et al. (2017), and Gao et al. (2023), respectively. Additionally, leaf organs, being the assimilation organs, tend to store C content. To meet growth requirements, plants transport substantial nutrients to branches, stems, and roots for storage in preparation for growth in the following year, and thus the leaf N content is higher in the growth phase than in the dormant phase. Zhang et al. (2022) showed that the N content allocated to leaves increases with altitude in Qilian Mountains. Furthermore, Qin et al. (2022) found a significant negative correlation between N content and N-to-P ratio in Qinghai spruce and temperature. These results suggests that high N and P content in leaf organs are beneficial for nutrient competition and represent an optimized resource allocation strategy “give-and-take” trade-off strategy (Zhang, 2004) that prioritizes leaves to meet photosynthetic needs. The utilization of plant nutrients and difference in nutrient distribution among organs at different growth stages are mainly influenced by the limited supply of environmental nutrients and the plant’s own physiological characteristics, as confirmed in the variance decomposition in **Table 2**. The K content in plants is related to their growth, drought resistance, and disease resistanc e. Plants with high K content exhibit strong resistance (Zhang et al., 2014). Our results show that the K content in all four forest types across different seasonal scales is lower than the K content reported by Wu et al. (2020) in the Maolan Karst forest (11.58 g/kg). During the growth phase, the K content in PA leaves (7.87 g/kg) is higher than than in PLL, AGS, and JS, but lower in PA trunk organs (0.57 g/kg). The possible reason is that leaf organs in the low-altitude PA area benefit from favorable hydrothermal conditions, which facilitate the accumulation of nutrients in the plant body. The trunk organs, serving as conduits between underground absorbing organs and aboveground assimilating tissues, have phloem and xylem that respond poorly to surrounding environmental changes (Meinzer et al., 2009). Hence, the K content is low. Furthermore, during the dormant phase, the K content in the JS leaves and branch organs is lower than that in PA leaves possibly due to the low nutrient demand for K content and high adaptability to the environment in JS leaf and branch organs.

Plants adapt to different stages of their living environment by allocating limited resources to different functional organs in certain proportions, forming specific growth characteristics and elemental distribution patterns (Li et al., 2016). Additionally, within the same forest type, the highest C content is observed in leaf organs across seasonal scales (**Figure 4A–D**). The seasonal variation in N content across the four forest types shows the order leaves > branches > roots > trunks mainly because leaf organs, as the site of photosynthesis, are carbon source organs that need to maintain a high metabolic rate and large production of C (Sala et al., 2012; Sala and Mencuccini, 2014). Branch organs play a role in nutrient transport and are the close to leaf organs, serving a supportive function by storing some nutrients while the leaves perform photosynthesis. Nutrients obtained by leaves from branches are consumed and converted into organic matter and then transported by branch organs to other parts. Thus, branches have higher nutrient content than roots and trunks, and leaves are the most metabolically active organs. This result is similar to the conclusion of Huang et al. (2021) but differs from that of Liu et al. (2016) possibly due to differences in species, which lead to variations in nutrient distribution among organs. Meanwhile, in the growth phase, the P and K content in PLL and AGS organs show the order branches > leaves > roots > trunks. During the dormant phase, the order is leaves > branches > roots > trunks in all the forest types. In summary, plants are influenced by the same environmental factors, such as climate and soil, showing a similar convergence in organ nutrient content (as shown in the N content of the study area). However, their genetic factors play a dominant role, resulting in differences in nutrient absorption and distribution among different forest types (as particularly evident in the P and K content). These findings are similar to those of previous research (Liu et al., 2015).

### 4.2 Seasonal dynamics of stoichiometric ratios of various organs in different forest types of plants

In natural plant communities, nutrient cycling is affected when the stoichiometric ratios of C, N, P, and K are imbalanced. Deficiency or excess of any one element can lead to the accumulation or depletion of others (McGroddy et al., 2004). Scarcity of N, P, and K implies an excessive C content; conversely, the abundance of N, P, and K indicates a relative deficiency in C (Güsewell, 2004). Thus, plant stoichiometric ratios can be used for assessing C accumulation and the limitation of N, P, and K nutrients. In this study, the C/N, C/P, and C/K ratios in the same organ in different forest types are significantly higher than the global plant nutrient ratios (22.50, 232.00, and 30.73) reported by Elser et al. (2000), indicating high N, P, and K utilization efficiency and C assimilation capacity in all four forest types throughout the seasons. High growth rates correspond to low C/N, C/P, and C/K ratios (Elser et al., 2000). During the growth phase, the lowest C/N ratios are observed in JS, and the highest ratio are observed in PA, suggesting that JS has a relatively lower nutrient utilization efficiency and higher growth rate. This result is consistent with the results of Li et al. (2013), who studied plants in the Baihua Lake drawdown zone and reported a survival strategy under nutrient-poor conditions. No significant changes were observed during the dormant phase.

Various factors, including climate conditions, plant species, soil characteristics, and growth stages, can affect the critical value of the N/P ratio. An N/P ratio <14 indicates N limitation in plant growth, whereas a ratio >16 suggests P limitation. When the N/P ratio is between 14 and 16, plant growth is limited by N and P (Zhang et al., 2023). In this study, the average N/P ratios in the leaves, branches, roots, and trunks of different forest types at various growth stages are all lower than 14, indicating N limitation in plant growth in this region. Across seasonal scales, JS has the highest N/P ratios for all organs, except the leaves, suggesting that organs in JS at higher altitudes have stronger N and P absorption capabilities than the organs in other forest types and has high resistance to adverse environments. According to Olde Venterink et al. (2003), when the N/K ratio is >2.1 and the K/P ratio is <3.4, K content is the main limiting factor. During the growth phase, the N/K ratios in the leaves of PLL (2.52) and AGS (2.86, 3.02) exceed 2.1, similar to the N/K ratios in the leaves, branches, roots, and trunks of JS. The other organs in different forest types are not limited by K content. Except the leaf organs in JS, the K/P ratio thresholds are above 3.4, indicating different nutrient limitation types during seasonal changes in different forest types. The plants in JS tend to be limited by K content.

Across different seasonal scales, the four forest types show similar convergent strategies in nutrient storage and utilization in various organs. The highest C/N, C/P, and C/K ratios are primarily found in trunks and roots, and the lowest are found in leaves and branches, indicating that trunks and roots in the four forest types of the study area have high N, P, K utilization efficiency and strong C assimilation capacities. The leaves and branches have low nutrient utilization efficiency for N and P during the same growth season and weak C assimilation capacities possibly because leaves function as photosynthetic organs requiring high N and P content for synthesizing various enzymes for biochemical reactions and C/N and C/P ratios are low in leaves. Meanwhile, leaf organs have the highest N/P and N/K ratios, suggesting that under N limitation, plants can minimize N limitation in leaves and thereby enhance metabolic production, consistent with adaptation strategies of plants in some alpine regions (Pang et al., 2020).

### 4.3 Response strategies of plant nutrients and ratios to environmental factors in different seasons

Different forest types reflect the ability of plants to adapt to external environments through physiological and biochemical processes. Regarding ecosystem interactions, a good correlation is observed among C, N, P, and K content, reflecting the convergent evolution of plants adapting to environmental changes (Sui et al., 1993; Niu et al., 2013). PCA further demonstrates (**Figures 6A, B**) that the ecostoichiometric traits of leaf organs show considerable clustering across different forest types with minimal variation, especially significant within the growth phase. As the most metabolically active organ, leaves prioritize stability in various environments, reducing sensitivity to environmental changes and ensuring carbon fixation and growth in plants. Moreover, plant carbon fixation capacity is influenced by multiple external environmental factors, such as solar radiation, temperature, humidity, water, and nutrient supply (Song et al., 2016). In this study, the ratios of C/N, C/P, and C/K show a highly negative correlation with N, P, and K content, which are highly positively correlated with one another. Additionally, AT and SH exhibit a highly positive correlation with C, N, P, and K content and with C/N and K/P ratios but a highly negative correlation with the C/K ratio. ST shows a highly positive correlation with N/P and N/K ratios. These findings indicate that the carbon sequestration ability of plants in the study area is minimally influenced by habitat, reflecting the synergistic variations in N, P, and K nutrients within the plants. This synergy is a critical factor for the stable growth and development of plants and represents one of their fundamental characteristics. These results are consistent with the findings of Wu et al. (2010) and Wang et al. (2014). Furthermore, during the growth phase, AT and SH significantly influence the ecostoichiometric ratios of various organs in PLL, and AT has a direct and considerable effect. In the dormant phase, ST significantly influences the ecostoichiometric ratios of JS organs. This variation may be attributed to different species and region-scale influences on plant nutrient absorption and demand. Additionally, as the elevation of forest stands increases, nutrient supply in the soil changes due to microbial activity, litter decomposition, environmental conditions, and plant uptake. In the growth and dormant phases, PC1 factor loadings reveal a clear separation of nutrient content and stoichiometric ratios along the PC1 axis. The trends in C, N, P, and K content is opposite to the trends in C/N, C/P, and C/K ratios, indicating that seasonal variations in the C/N, C/P, and C/K ratios of plant organs are primarily governed by N, P, and K, consistent with the growth rate hypothesis.

## 5 Conclusions

Influenced by environmental factors, such as forest type and altitude, different forest types exhibit variations in nutrient absorption and utilization efficiency in their organs. The study area is characterized by high C but low N, P, and K content. The CV in nutrient content across organs indicates a tendency for leaf organs to maintain stable carbon content. Hence, we infer that active organs have a robust capacity to maintain a stable elemental content and ratio through a “conservative” strategy. The increase in N content in plant leaves at high altitudes represent an active adaptation of plants, consistent with the temperature–plant physiological hypothesis. Owing to the influence of seasons, fluctuations in the evolution process of N and P content lead to an optimized allocation of nutrients toward support and transport organs. The intercoupling of plant nutrients C, N, P, and K reflects a high level of complexity in the synergy of changes in nutritional element requirements within plants. Overall, different forest types exhibit various nutrient limitation types, primarily limited by N and P, with a greater susceptibility to N limitation. Therefore, in the management of the Sygera Mountain forest area, fertilization strategies should consider these forest type differences.

## Data availability statement

The original contributions presented in the study are included in the article/Supplementary Material. Further inquiries can be directed to the corresponding author.

## Author contributions

YL: Conceptualization, Formal analysis, Methodology, Writing – original draft. JW: Funding acquisition, Investigation, Software, Writing – original draft. LW: Data curation, Validation, Visualization, Writing – review & editing.
